# A new era in oral treatment for acromegaly with the FDA-approved drug paltusotine

**DOI:** 10.1097/MS9.0000000000004426

**Published:** 2025-12-03

**Authors:** Suhail Ahmed, Hayat Asghar, Muddassir Khalid

**Affiliations:** a Department of Medicine, Liaquat University of Medical & Health Sciences (LUMHS), Jamshoro and Pakistan; b Department of Medicine, Nishtar Medical University, Multan, Pakistan

*Dear Editor,*

Paltusotine was approved by the U.S. Food and Drug Administration (FDA) on 26 September 2025, as the first once-daily oral treatment for acromegaly in adults for whom surgery was either ineffective or not an option[1].This approval marks an important milestone in the management of acromegaly, a chronic hormonal disorder requiring long-term medical therapy. A benign pituitary adenoma typically causes acromegaly, a rare endocrine disorder that results in excessive growth hormone (GH) secretion and elevated insulin-like growth factor-1 (IGF-1) levels. Paltusotine’s approval represents a significant step forward in the long-term medical treatment of acromegaly, particularly for patients switching from injectable somatostatin analogs to oral therapy. Even though injectable somatostatin analogs are effective, the need for more convenient oral therapies has been brought to light by their lifetime administration requirements and related discomfort. Injectable somatostatin analogs like octreotide or lanreotide have long been used to treat acromegaly, which is characterized by elevated IGF-1 and chronic GH hypersecretion. It is anticipated that the creation of a successful oral substitute will significantly increase patient compliance, lessen the burden of treatment, and improve quality of life. A nonpeptide selective somatostatin receptor-2 agonist called paltusotine is being developed as a once-daily oral medication to treat acromegaly. In patients with acromegaly, replacing injected somatostatin receptor ligands (SRLs) with once-daily oral paltusotine was well tolerated and maintained both biochemical and symptom control[2]^.^

Paltusotine’s therapeutic benefit stems from both its distinct molecular mechanism and oral formulation. By selectively binding to the somatostatin receptor subtype 2 (SSTR2), paltusotine, an oral small-molecule agonist, activates Gi protein-coupled signaling and inhibits adenylyl cyclase, thereby lowering intracellular cAMP levels. GH and IGF-1 secretion are suppressed by this action. According to cryo-EM research, paltusotine produces a unique SSTR2 conformation that leads to biased signaling and enhanced receptor selectivity, offering a targeted and efficient treatment for neuroendocrine tumors and acromegaly. Its clinical superiority is further elucidated by comprehending the pharmacologic differences between this novel nonpeptide agent and conventional peptide analogs. Although both paltusotine and octreotide work via SSTR2, their mechanisms vary in terms of selectivity and efficiency. Compared to octreotide, paltusotine exhibits a more effective and selective mechanism. While octreotide, a first-generation peptide, has variable cell surface expression and limited receptor subtype selectivity, which can result in poorer responses in certain patients, it activates SSTR2 through a unique, biased signaling pathway that guarantees stronger therapeutic effects with fewer side effect[3]. .

Paltusotine, a once-daily oral nonpeptide SSTR2 agonist, demonstrated a long half-life (~30 hours), good oral bioavailability, and dose-dependent suppression of GH (44%–93%) and IGF-1 (19%–37%). As a novel, practical oral option for the treatment of acromegaly and neuroendocrine tumors, it was well tolerated and had a safety profile comparable to injectable SSAs[4]

In addition to its effectiveness, paltusotine’s safety and tolerability profile have shown promise in clinical trials. Paltusotine was well tolerated in the PATHFNDR-2 trial, and no significant side effects were noted. Headache, nausea, and exhaustion were the most frequent adverse effects, which were mild and typical of SRLs or acromegaly symptoms. During the study, no unexpected or novel safety signals were found[5] .Paltusotine’s FDA approval represents a major advancement in the treatment of acromegaly. By doing away with the need for injectable treatments, this first once-daily oral somatostatin receptor agonist has the potential to revolutionize existing treatment approaches and increase patient compliance. However, additional long-term research is necessary to completely determine its long-term effectiveness, safety, and function in more comprehensive endocrine indications.

This manuscript is compliant with TITAN Guidelines, 2025, declaring no use of AI^[6]^.

## Data Availability

Not applicable.

## Peer and provenance statement

Not commissioned.
